# A Bayesian approach for identifying miRNA targets by combining sequence prediction and gene expression profiling

**DOI:** 10.1186/1471-2164-11-S3-S12

**Published:** 2010-12-01

**Authors:** Hui Liu, Dong Yue, Lin Zhang, Yidong Chen, Shou-Jiang Gao, Yufei Huang

**Affiliations:** 1 SIEE, China University of Mining and Technology, Xuzhou, China; 2 Department of Electrical and Computer Engineering, University of Texas at San Antonio; 3 Department of Pediatrics, University of Texas Health Science Center at San Antonio; 4 Greehey Children's Cancer Research Institute, University of Texas Health Science Center at San Antonio; 5 Department of Epidemiology and Biostatistics, University of Texas Health Science Center at San Antonio

## Abstract

**Background:**

MicroRNAs (miRNAs) are single-stranded non-coding RNAs shown to plays important regulatory roles in a wide range of biological processes and diseases. The functions and regulatory mechanisms of most of miRNAs are still poorly understood in part because of the difficulty in identifying the miRNA regulatory targets. To this end, computational methods have evolved as important tools for genome-wide target screening. Although considerable work in the past few years has produced many target prediction algorithms, most of them are solely based on sequence, and the accuracy is still poor. In contrast, gene expression profiling from miRNA transfection experiments can provide additional information about miRNA targets. However, most of existing research assumes down-regulated mRNAs as targets. Given the fact that the primary function of miRNA is protein inhibition, this assumption is neither sufficient nor necessary.

**Results:**

A novel Bayesian approach is proposed in this paper that integrates sequence level prediction with expression profiling of miRNA transfection. This approach does not restrict the target to be down-expressed and thus improve the performance of existing target prediction algorithm. The proposed algorithm was tested on simulated data, proteomics data, and IP pull-down data and shown to achieve better performance than existing approaches for target prediction. All the related materials including source code are available at http://compgenomics.utsa.edu/expmicro.html.

**Conclusions:**

The proposed Bayesian algorithm integrates properly the sequence paring data and mRNA expression profiles for miRNA target prediction. This algorithm is shown to have better prediction performance than existing algorithms.

## Background

MicroRNAs (miRNAs) are single-stranded non-coding RNAs with about 19 to 25 nucleotides in length. MiRNA is known to inhibit target translation or cleave target mRNA by binding to the complementary sites in the 3’ untranslated region (UTR) of targets. The importance of miRNA regulation lies in the fact that a miRNA is estimated to regulate hundreds of targets [1]. As a result, miRNAs have been shown and are speculated to play many important post-transcriptional regulatory roles in a wide range of biological processes and diseases including development, stress responses, viral infection, and cancer [2-5]. Despite rapid advance in miRNA research, the detailed functions and regulatory mechanisms of most of miRNAs are still poorly understood. To gain better understanding, an important task is to identify miRNAs’ regulatory targets. However, the current knowledge about the known targets is disproportional to that of the known miRNAs. In the miRNA registry miRBase, 969 human miRNAs are annotated; in contrast, only 815 targets of 121 human miRNAs are recorded in the most up-to-date target database miRecords. Given that the number of targets of each miRNA could be in hundreds [1], the reported number of verified targets accounts for only a very small fraction of the potential human targets. This fact greatly underscores the urgent need of effective target identification methods, and, for genome-wide target discovery, computational prediction proceeding experimental testing is a preferable, efficient strategy. Considerable advances have been made in computational target prediction [6] and many prediction algorithms have been proposed, mainly based on various important features of miRNA:target nucleotide sequence interaction. Although different algorithms utilize different sets of features, a few important features including “seed region complementary”, “binding free energy”, and "sequence conservation" are among the most common ones. Depending on how these features are derived, the algorithms using sequence binding data can be further categorized into the rule based and the data driven. In the rule-based algorithms, features are determined from the prior knowledge of miRNA binding and these algorithms include TargetScan [7], miRanda [8], PITA [9], DIANA-microT [10], RNAhybrid [11], microInspector [12], MovingTargets [13], and Nucleus [14]. In contrast, for the data driven algorithms, the features are partially or entirely determined by the algorithm itself from the training data, or the existing sequence binding data of verified positive and negative miRNA:target pairs. The data driven algorithms include MirTarget [15,16], PicTar [17], miTarget [18], rna22 [19], NBmiRTar [20], Targeting [21] and SVMicrO [22]. Given sufficient training data, the data driven algorithms hold the promise to outperform the rule based algorithms, since they have the ability to uncover important features from data that cannot be easily observed otherwise.

Despite these effort, the existing algorithms using sequence data alone are still of poor prediction specificity and sensitivity [23,24]. The first reason of the deficient performance is due to the poor understanding of the precise mechanisms underlying miRNA:target interaction [25-27] and, as a result, the adopted features of the rules are not yet as specific and sensitive as needed. Secondly, verified positive and negative training data essential for good performance of data driven algorithms are particularly lacking and the limited verified data can hardly include important features for different aspects of the miRNA:target interactions, thus hampering the ability of date driven algorithms to select discriminative features[28]. These facts motivated us to incorporate data other than sequence pairing to further improve the prediction performance of existing algorithms.

Microarray profiling of differential gene expression after miRNA transfection is a widely adopted approach to investigate the impact of the miRNA regulation. Such gene expression profiles have been used in a variety of studies for predicting miRNA targets. However, the majority of existing research relies on the assumption that miRNA targets are down-expressed in microarray and thus search within the intersection of sequence level prediction and down-regulated genes in microarray for potential targets[29,30]. Given that the primary function of miRNA is translation inhibition with target mRNA degradation being the secondary mode of regulation, the down-expression of mRNA is neither the sufficient nor the necessary condition for miRNA regulation. Therefore, the outcome of this practice is unlikely to greatly reduce the high false positive rate; on the contrary, it deteriorates more the prediction sensitivity.

**Figure 1:**
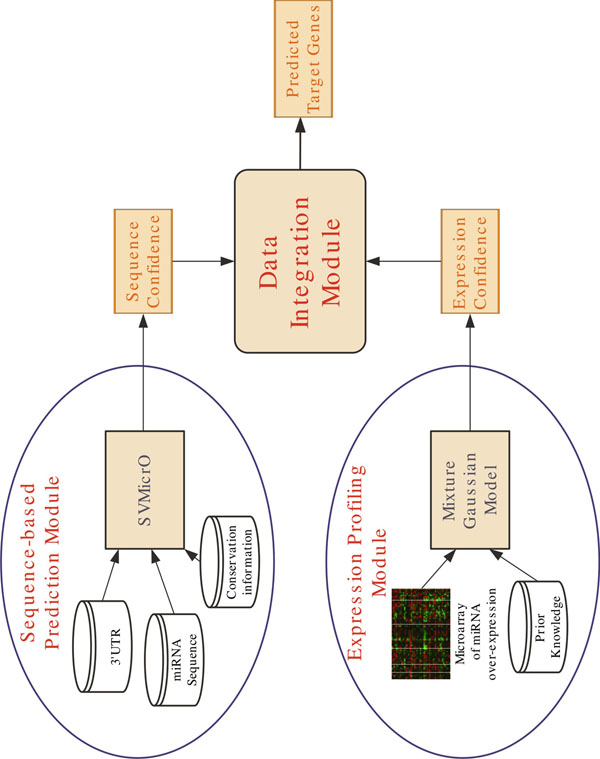
**Algorithm Block Diagram** The proposed algorithm consists of a sequence-based prediction module and a expression profile inference module. A Naïve Bayes model integrates the outputs of these two modules to generate final prediction score.

To address the problem with the current practice in combining sequence prediction with microarray data, we present a novel Bayesian algorithm with the scheme shown in Figure 1. In particular, a Bayesian Gaussian Mixture Model (GMM) is applied to model the expression profile of positive and negative targets. This model allows not only the positive targets to be not differentially expressed but also the negative targets to be down-expressed. In particular, to properly model the mixture component for positive targets, the prior distribution constructed based on the existing expression profile of real targets is introduced. Consequently, this model can describe the realistic distribution of positive and negative miRNA target expression. Finally, the probability of an mRNA as a target given the mRNA expression and the prediction score of its corresponding sequence binding are integrated by a Naïve Bayes model. The algorithm is applied to predict targets of hsa-miR-1 and hsa-miR-124, and the prediction performance is evaluated by the IP pull-down and mass spectrometry experiments. The results show the improved performance of the proposed algorithm for miRNA target prediction.

## Methods

### Problem statement

For convenience of composition, the mathematical definition of the problem is first given. For a given mRNA *g* and a given miRNA m, let *t* ∈ {0, 1} denote whether *g* is a target of m. Let *S* indicate the sequence paring information of *g* and *m*. Let *e* represent the differential expression (log fold change) of *g* due to transfection of miRNA m. The goal of target prediction is to select most possible value of *t* base on the expression *e* and sequence paring *S.* According to a Naïve Bayes formulation, the desired *a posteriori* probability (APP) can be calculated as shown in formula (1)(1)

where the second equality is arrived based on the assumption that *e* and *S* are independent, and *α*(*e*) and *β*(*S*) are the APPs of *t* given *e* and *S,* respectively. Although *e* and *S* are not independent in reality, this assumption reduces the complexity of modeling and the subsequent computation. Additionally, the Naïve Bayes formulation has been shown to be able to achieve satisfactory performance even when the data are correlated. We will discuss next the models and approaches for calculating *α*(*e*) and *β*(*S*)*,* respectively.

### Mapping of sequence level prediction scores to *β*(*S*)

There exist several target prediction algorithms using sequence data. We adopt our own SVMicrO algorithm in the work since it has been shown to outperform other popular algorithms. Like most of target prediction algorithm, SVMicrO produces a score *s* for each miRNA:mRNA sequence pairing to indicate the confidence of the mRNA to be a target. To obtain *β*(*S*) from SVMicrO score *s*, SVMicrO score *s* is assumed to contain all the information of the sequence *S* and *β*(*S*) can be therefore calculated as *p*(*t =* 1|*s*) instead of *p*(*t = 1|S*)*.* The goal is then to map the score into the APP *β*(*s*) *= p*(*t =* 1*|S*)*.* To this end, a logistic model is used as(2)

where *α*_0_ and *α*_1_ are the parameters to be trained.

### Training *β*(*s*)

**Figure 2:**
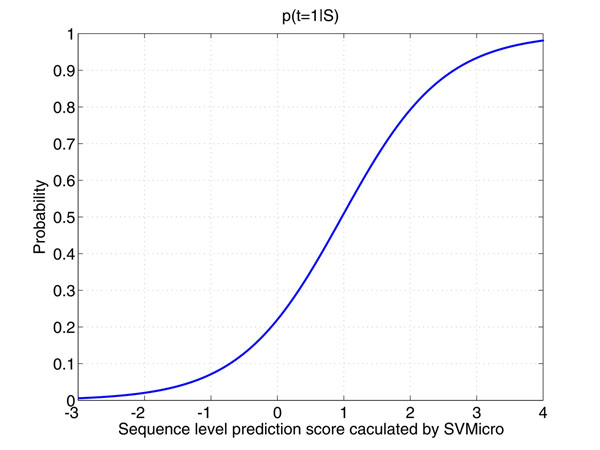
Curve of *β*(*S*) modeled by the Logistic Regression Function

The training data used for training SVMicrO were adopted here to train *β*(*S*)*.* In brief, the training data set is composed of 509 experimental validated miRNA:Target pairs recorded in miRecords [1] and 2426 high confidence negative miRNA:Target pairs derived from microarray data sets of 20 different miRNA transfection experiments (See Table 1). SVMicrO was then trained by a 5-fold cross validation; the average predicted scores of each gene in the training data were obtained. These scores together with their associated target attributes were used as training data for estimating the parameters of the logistic function *β*(*s*)*.* The curve of trained *β*(*s*) is shown in Figure 2. It can be noticed from Figure 2 that the probability of a mRNA to be a target is only around 50% even if the predicted score is 1. This demonstrates the inability of sequence-based approach to achieve satisfactory precision; this problem is partially due to the huge imbalance between positive and negative data.

**Table 1:** Microarray Data Source of Negative Samples

miRNA	GEO accecsion	miRNA	GEO accecsion
hsa-let-7c	GSM156557[33],GSM156558[33]	hsa-miR-128	GSM210902[7],GSM210903[7]

hsa-miR-15a	GSM156545[33],GSM156549[33]	hsa-miR-132	GSM210904[7],GSM210905[7]

hsa-miR-16	GSM156546[33],GSM156550[33]	hsa-miR-133a	GSM210906[7],GSM210907[7]

hsa-miR-17	GSM156553[33],GSM156555[33]	hsa-miR-142-3p	GSM210908[7],GSM210909[7]

hsa-miR-192	GSM156547[33],GSM156551[33]	hsa-miR-148b	GSM210910[7],GSM210911[7]

hsa-miR-20a	GSM156554[33],GSM156556[33]	hsa-miR-7	GSM210896[7],GSM210897[7]

hsa-miR-215	GSM156548[33],GSM156552[33]	hsa-miR-9	GSM210898[7],GSM210899[7]

hsa-miR-192	GSM328290[34],GSM328287[34]	hsa-miR-34a	GSM187633[35],GSM187634[35]
			GSM187631[35],GSM187632[35]

hsa-miR-215	GSM328291[34],GSM328288[34]	hsa-miR-34b	GSM190765[36],GSM190757[36]

hsa-miR-122	GSM210900[7],GSM210901[7]	hsa-miR-34c-5p	GSM190758[36],GSM190766[36]

### Gaussian mixture models of expression profile

**Figure 3:**
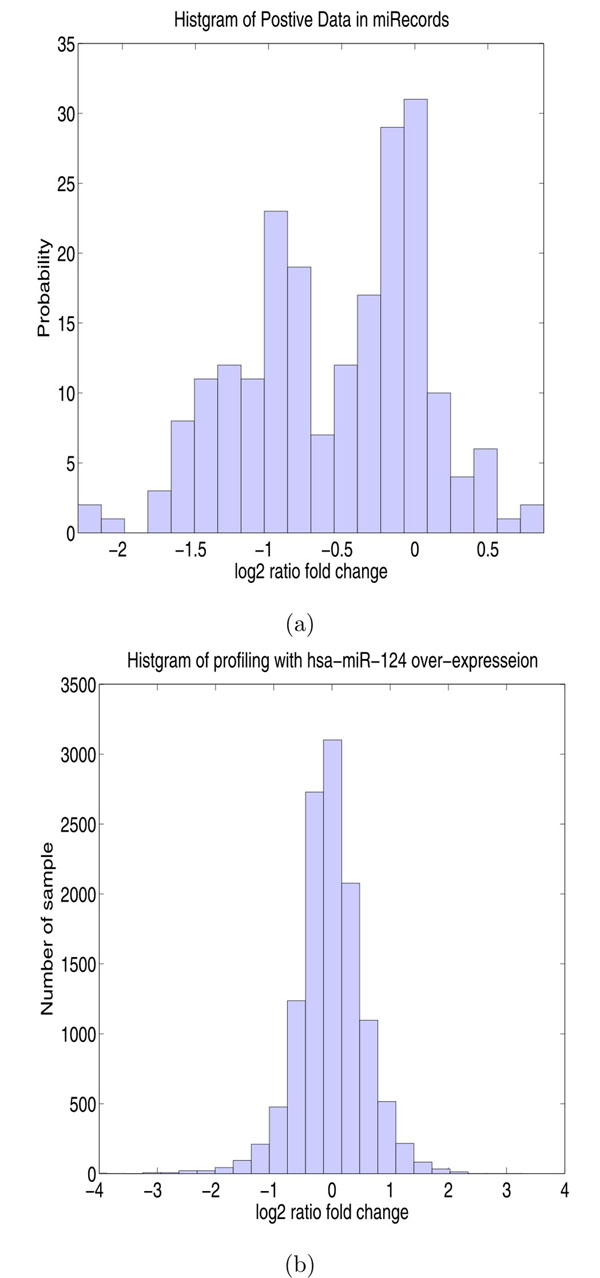
**Histograms of Gene Expression Profiles***(a) Histogram of the expression fold change of 209 verified targets in different miRNAs transfection experiments. Notice that a portion of the true targets were not down-regulated. (b) Histogram of genome-wide expression of hsa-miR-124 transfection* The genome-wide expression profile consists of those for both positive targets and negative targets. Since positive targets for a small portion of the genome, the distribution of genome expression looks like the distribution of the negative targets.

The gene expression profile of miRNA transfection experiment contains both the expressions of the positive as well as negative targets, both of which needs to be properly modeled. To this end, the empirical distributions of expression was first examined. To obtain the expression of verified targets, the verified targets of human miRNAs recorded in miRecords [1], a depository for experimentally verified miRNAs targets, were obtained first. The expression fold change of each recorded target was retrieved whenever the corresponding miRNA transfection experiment is registered in GEO. Finally, fold change value of 209 verified targets were obtained and the histogram of the their expression fold change is depicted in Figure 3-(a). For computational convenience, expression data for both positive and negative data are assumed to be the Gaussian distributions. Therefore, the genome-wide expression data is modeled as a mixture Gaussian distribution(3)

where *μ*., *σ*^2^ are the mean and variance of the respective Gaussian mixtures, the subscripts + and — denote the positive (*t* = 1) and negative (*t* = 0) targets, *π*_+_ + *π* = 1, and ***θ*** represents the collection of the model parameters. Given model (3), the goal is to uncover mixture components from the expression data, which is equivalent to estimate the parameters from the expression data. Note that since the number of positive targets is only in hundreds, *π*_+_ is very small, which means that the component of the positive target is much weaker compared with the negative target and likely to be completely buried in the mixture. This can be illustrated by Figure 3-(a), where the histogram of genom-wide expression of 11988 human mRNAs for transfection of hsa-miR-124 [31] is plotted. Since the true targets of a miRNA counts for only very small portion of the entire genome, the histogram of the genome-wide expression for transfection of hsa-miR-124 appears more like a single Gaussian instead of a mixture of two. Unless additional information about the expression of positive data is available, the estimation of the positive component from the mixture is under-determined and there could be a large number of suboptimal solutions. Fortunately, the expression data of experimentally validated targets are available. These expression levels, although limited in quantity, can be used to aid the estimation of the positive component. which Supposedly,

### Bayesian estimation of the gaussian mixture

Under the Bayesian framework, the goal of estimating model parameters ***θ*** is to obtain the posterior distribution

*p*(***θ***|*e*) ∞ *p*(*e*|***θ***)*p*(***θ***) (4)

where *p*(****θ***|e*) is the likelihood defined in (3) and *p*(***θ***) is the parameter prior distribution. Here, the conjugate priors are adopted and a combination of informative and noninformative priors are defined as(5)

where *NIG* and *Dir* are the Normal-Inverse-Gamma and Dirichlet distributions, respectively and **e**p**denotes the expression profile of the validated targets. It should be clear that an informative prior is applied for the positive component, whereas the noninformative prior is imposed to the negative component. We discuss next the details of these priors. First, the informative *NIG* prior of  can be obtained from **e***p* using the standard Bayesian linear Gaussian model by applying a Gaussian likelihood and another noninformative *NIG* prior. Specifically, given the prior of *μ_+_* and  follows the noninformative *NIG* distribution(6)

the informative can be shown to be(7)

where(8)

*N =* 209 in our case, *ē_p_* and *s*^2^ are the sample mean and variance of **e_p_**, and all other parameters with subscript 0 are the same as those in (5), which define the noninformative prior. Next, for the noninformative priors in (5) and (6), the parameters are chosen as:

*μ*_ = 0, *σ*_ = 5, *μ*_0_ = 0, *κ*_0_ = 0.2, *α*_0_ = 0.2, *β*_0_ = 0.2.

Lastly, the parameters of the Dirichlet prior are chosen as *γ*_+_,0 = 200 and *γ*_,0 = 20000, which reflects the common belief that a miRNA regulates about 200 targets.

Since the likelihood assumes the mixture model in (3), the posterior distribution cannot be obtained analytically. A Variational Bayes Expectation Maximization (VBEM) algorithm is applied to estimate the desired distributions.

### Variational bayes expectation maximization algorithm

Since the expression level of each gene is assumed to be i.i.d. and follows the Gaussian mixture (3), the parameters should be estimated from the gene expression profile of all genes ***e*** = {*e*_1_, ⋯, *e_G_*}. VBEM algorithm starts by constructing a lower bound on the marginal likelihood function as(9)

where as above the inequality is due to the Jensen's inequality, , as well as *q*(*π*) and *q*(*ϕ*) are the free distributions introduced to approximate the unknown posterior distributions *p*(*π|e*) and *p*(*ϕ|e*)*.* The distributions *q*(·) (or their parameters) are determined to maximize the lower bound (9). Using the variational derivatives and an iterative coordinate ascent procedure, the optimization can be achieved in an iterative fashion, whose *j* + 1 iteration operates as follows:

VBE Step:(10)

**Figure 4:**
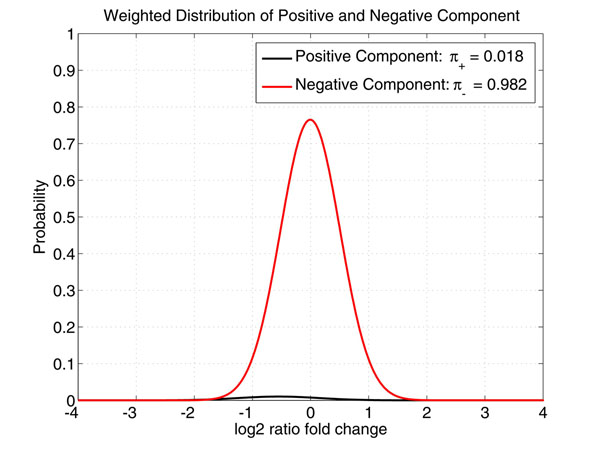
**Weighted Distributions of Positive and Negative Components with Parameters Estimated from Data** The parameters of both positive and negative are estimated by the VBEM algorithm.

VBM Step:(11)

where *Z*(·)*s* are the normalizing constants. Since *q*(*π*) and *q*(*ϕ*) are assumed to be the Dirichlet and *NIG* distributions, (10) and (11) can be obtained analytically. Then, when the algorithm converges, we obtain the approximations to the distributions *p*(*π|e*) and *p*(*ϕ|e*) as *q*(***π***) and *q*(*ϕ*)*,* respectively. The MAP or MMSE estimates of *π* and *ϕ* can be obtained from *q*(*π*) and *q*(*ϕ*) accordingly. An example of the estimated mixture distributions weighted by *π* is shown in Figure 4.

### Calculation of *α*(*e*)

**Figure 5:**
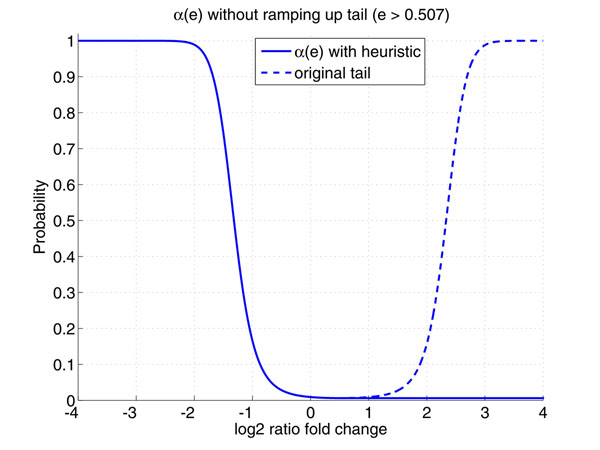
**Curve of *α*(*e*) Obtained from the Gaussian Mixture Model from Real Data** The curve of *α*(*e*) is monotone decreasing in the area of [-4, 0.5], which agree with the existing knowledge. However, the curve unexpectedly ramps up (the broken line)due to the heavier tail of the positive component. To resolve this problem, we simply fix the curve to be a constant. This heuristic is simple but works well in practice. The solid line in Figure 4 visualizes *α*(*e*) with this heuristic

With the estimated parameters, *α*(*e*) can be calculated as(12)

where ^ represents the estimate of the corresponding parameter. Based on the parameters estimated by VBEM algorithm, *α*(e) can be fully defined by (12) and its curve is plotted as Figure 5. The curve is monotone decreasing in the area of [-4, 0.5], which reflects the existing knowledge that the more significant that an mRNA is down-regulated in the miRNA transfection experiment, the more likely the mRNA is a real target. However, the curve ramps up (broken line) afterwards due to the higher tail of the positive Gaussian component. This phenomenon does not agree with the fact that the higher the expression fold change, the unlikely the mRNA is a target. To resolve this problem, we simply fix *α*(*e*) as the constant for expression fold change larger than 0.5. This heuristic is simple but works well in practice. The solid line in Figure 5 visualizes *α*(*e*) with this heuristic.

## Results and Discussion

### Validation Based on Simulated Data

We first tested the proposed algorithm based on the simulated data set. Particularly, we generated the sequence level prediction scores of both positive and negative data from two Gaussian distribution, whose means and variances were chosen based on the prediction scores of SVMicrO on the real positive and negative targets. The expression fold change data were produced from the Gaussian Mixture Model; the parameters of mixture model were chosen also based on those fitted to the expression fold changes of real positive and negative targets. To also reflect the imbalance between the positive and negative targets, 200 positive data and 19800 negative data were generated with distributions shown in Table 2.

**Table 2:** Distributions and parameters used to generate test data

	sequence score	fold change	mixture coefficient
Positive	*N*(0.75, 0.5)	*N*(–0.5, 0.5)	1%

Negative	*N*(–0.75, 0.5)	*N*(0, 0.4)	99%

Fitting of function *α*(e) is the most demanding process in this algorithm, especially due to the large imbalance in the two mixture components. As such, the ability of VBEM to accurately estimate the parameters of the GMM model is evaluated. The estimated parameters for the simulated data are shown in Table 3 and the weighted distributions of positive and negative components are shown in Figure 5. From Table 3, it can be seen that the VBEM algorithm succeeded in correctly estimating the parameters (see Table 2) used to generate the testing data.

**Table 3:** GMM parameters estimated by VBEM

	fold change	mixture coefficient
Positive	*N*(–0.4714, 0.5573)	1.8%

Negative	*N*(0.0044, 0.3994)	98.2%

**Figure 6:**
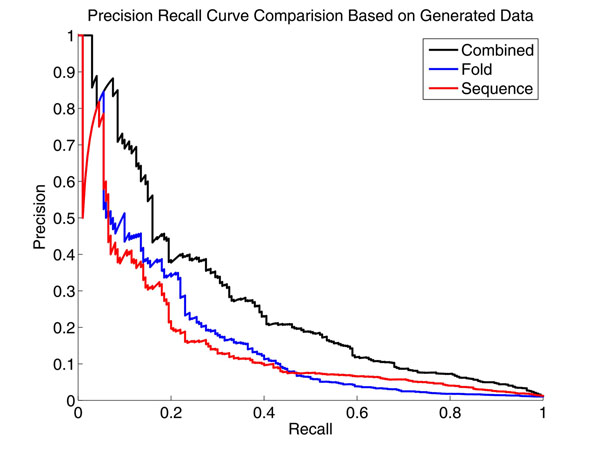
**Precision Recall Curve Comparison Based on Simulated Data** This figure indicated that the performance of proposed algorithm is better than those using either sequence information or expression data alone.

Next, precision recall curve was plotted to compare the performance of combined method with algorithms only relying on either sequence level score or expression fold change. Precision represents the odds of a predicted target to be the true target, while recall denotes the chance of having predicted the entire true targets. High precision often concerns biologists more because it is highly desirable and efficient to allocate the limited resource to test a set of predictions with high chance to be the true targets. However, recall is also important to assure that all the true targets can be uncovered. Overall, the larger the area under the PR curve an algorithm has, the better it is. As can be seen from Figure 6, the proposed algorithm has both better precision and recall and it achieves the overall best performance. Therefor, we can draw the conclusion that the performance of the combined algorithm improves the algorithm that relies on either sequence level data or expression fold change.

### Evaluation on real data

The proposed algorithm was applied to predict the targets of hsa-miR-1 and hsa-miR-124. The result was validated by the mass spectrometry data in [32] and the IP pull-down data in [31].

### Sequence Score and Differential Expression Data Retrieval

3'UTR sequences of human genome were downloaded from UCSC Genome Browser mySQL database. Prediction of genome-wide targets of hsa-miR-124 and hsa-miR-1 based on the sequence pairing data were carried out by SVMicrO. The prediction scores were recorded for each mRNA, which were then mapped to the APPs of being targets using the logistic function *β*(*S*) defined in (2). Gene expression profile of transfecting hsa-miR-124 or hsa-miR-1 was obtained from [31] and the APPs of targets given expression fold changes were calculated based on the function *α*(*e*) defined in (12) with heuristics. The integrated score was calculated based on (1) as a product of *β*(*S*) and *α*(*e*)*.*

#### Evaluation using Mass Spectrometry Data

**Figure 7:**
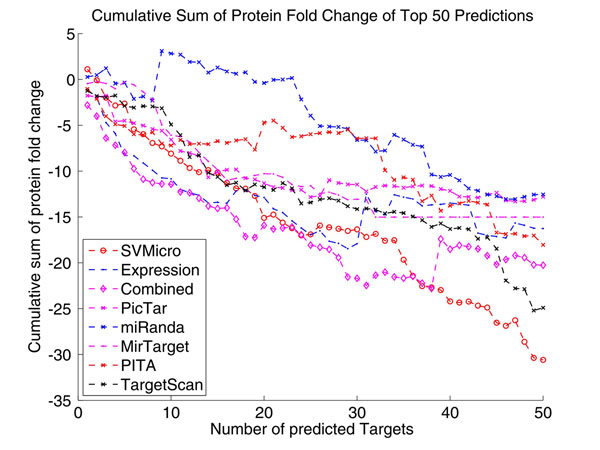
**Cumulative Sum of Protein Fold Change of Top 50 Predictions of hsa-miR-124** This figure shows the result for the top 50 predictions, which indirectly reflects the prediction precision. Particularly, the approach "Expression" uses simply mRNA expression as a score and ranks the larger down-expressed gene higher in the list. We note from Figure 7 that the proposed approach (Combined) achieves the highest amount of protein level down-fold for the top 35 predictions, which indicates higher precision of the proposed approach.

**Figure 8:**
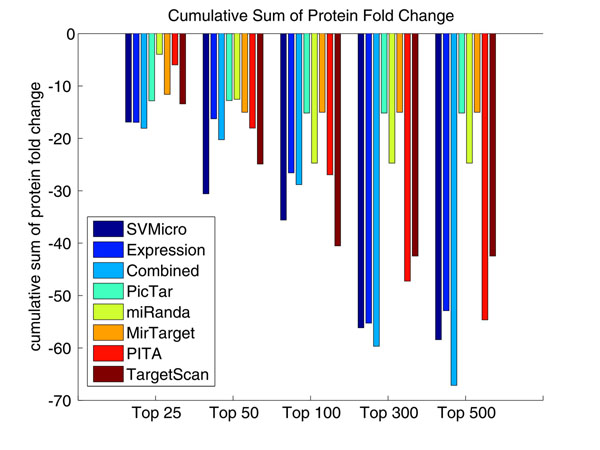
**Cumulative Sum of Protein Fold Change for Different Number of Top Ranked Predictions of hsa-miR-124** The cumulative sum of different numbers of top predictions for several algorithms are depicted. This figure shows that, after top 50, the proposed algorithm has the largest down fold, which also suggests higher sensitive for the proposed algorithm.

**Figure 9:**
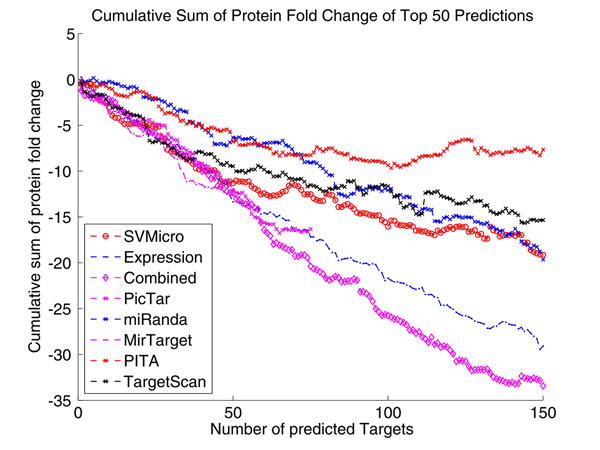
**Cumulative Sum of Protein Fold Change of Top 150 Predictions of hsa-miR-1** We note similar superior performance of the proposed approach as in Figure 8.

**Figure 10:**
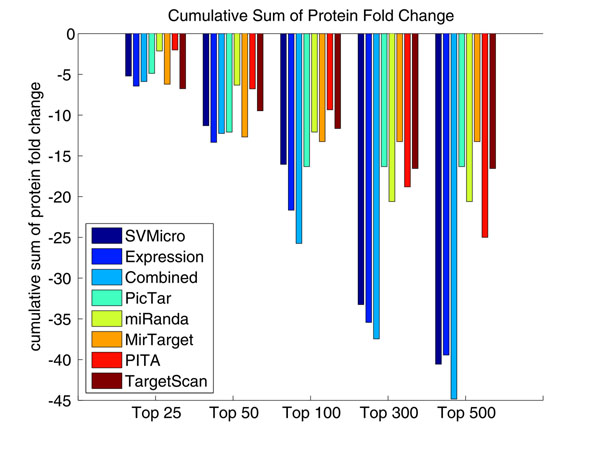
**Cumulative sum of protein fold change for different number of top ranked predictions of hsa-miR-1** The cumulative sum of different numbers of top predictions for several algorithms are depicted. This figure shows that, after top 100, the proposed algorithm has the largest down fold, which also suggests higher sensitive for the proposed algorithm.

To evaluate the performance, we first consulted the proteomics data of [32], which measure the protein level of differential expression derived from transfecting hsa-miR-124 or hsa-miR-1. Since protein inhibition is the primary mode of miRNA silencing, the protein level down-expression should be correlated more directly to the targets than mRNA expression level. As a result, it is of higher confidence to consider the proteins larger down fold as real targets. The data consist of the fold change of 1521 proteins. Intuitively, a better prediction algorithm should have higher down-expressed proteins among the top of the prediction ranked by the score. Accordingly, we ranked the prediction according to the scores calculated by each investigated algorithms and then examine the cumulative sum of their protein down-regulation in the ranked predictions. Figure 7 shows the result for the top 50 predictions for hsa-miR-124, which indirectly reflects the prediction precision. Particularly, the approach "Expression" uses simply mRNA expression as a score and ranks the larger down-expressed gene higher in the list. We note from Figure 7 that the proposed approach (Combined) achieves the highest amount of protein level down-fold for the top 35 predictions, which indicates higher precision of the proposed approach. The results of different numbers of top predictions for several algorithms are further depicted Figure 8. After top 300, the proposed algorithm has the largest down fold, which also suggests higher sensitive of the proposed algorithm. The same test was implemented for hsa-miR-1, and the similar results are shown in Figure 9 and Figure 10. We conclude based on these results that the proposed algorithm outperforms the sequence-based prediction and the prediction based expression data alone.

#### 0.0.1 Precision-Recall (PR) Performance using IP pull-down data

**Figure 11:**
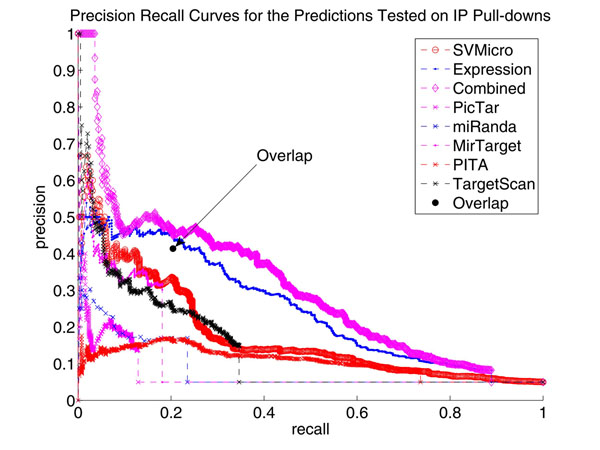
**Precision Recall Curves for the Predictions Tested on IP Pull-downs of hsa-miR-124** This figure shows a clear enhancement in both precision and recall compared to SVMicrO, the approach using expression data, and other sequence-based prediction algorithms. Besides, the overlapping method (black dot) only improves the precision slightly compared to SVMicro but is much worse our compared with the proposed algorithm.

**Figure 12:**
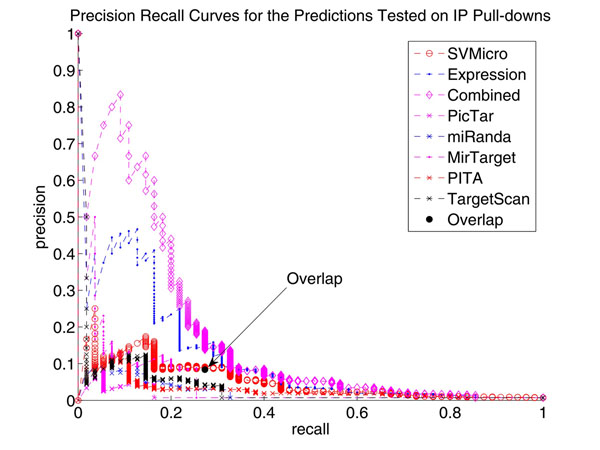
**Precision Recall Curves for the Predictions Tested on IP Pull-downs of hsa-miR-1** This figure shows again the similar performance improvement as Figure 11.

Since the utility of the evaluation on proteomic data is limited by the coverage of the SILAC technology and the potential noise in protein quantification, we further validated the prediction of hsa-miR-1 and hsa-miR-124 using the Immunoprecipitation (IP) pull-down data (Hendrickson, et al., 2008), which measures the potential targets recruited by the ARG-2, an important component of the miRNA effector protein complexes. In this experiment, 59 and 388 genes were determined as high confidence targets of hsa-miR-1 and hsa-miR-124, respectively, at a stringent FDR level of 0.01. We then treated these genes as the true targets and investigated the PR performance of different algorithms. The Precision-Recall curve of the proposed algorithm as well as SVMicrO, expression fold change, PicTar, miRanda, MirTarget, PITA and Target Scan were plotted as Figure 11 and Figure 12. The result shows a clear enhancement in both precision and recall of the proposed approach when comparing other tested algorithms.

### Comparison with the Overlap Method

As we mentioned before, most literature considers overlapping between sequence level prediction and down-regulated mRNA for target prediction. The performance of such overlapping scheme was also evaluated. In Figure 11 and Figure 12, the black dotindicates the precision and recall of the method that considers the intersection of SVMicrO prediction and down-regulated mRNA as targets. First, this overlapping method is outperformed by the proposed combined method. Secondly, it can be noted that the performance of this is not consistent. Particularly, for hsa-miR-124, the performance is slightly improved compared to SVMicro, while for hsa-miR-1 the performance greatly deteriorates. By investigating the detailed prediction results, we found that some of the experimentally validated targets were not down-regulated but predicted as positive by SVMicrO. Examples include NM080430, NM001078174, NM144706, NM001040402 and so on for hsa-miR-124 and NM002622 for hsa-miR-1. These positive predictions by SVMicrO were reverted to negative by the overlapping approach. This is the very reason why the precision cannot be increased. Therefore, a conclusion can be drawn once more that searching down-regulated mRNAs for targets is not an effective approach. Our proposed method provides a proper model for the true distribution of miRNA targets. As a result, improved performance can be achieved.

## Conclusions

In this paper, we presented a novel algorithm for miRNA target prediction by integrating sequence level prediction results with microarray expression profiling of miRNA transfection. A Gaussian mixture model was designed to model the gene expression profiles of the positive and negative targets and a Bayesian algorithm is devised to integrate the data. The validation results on both proteomics and IP pull-down data demonstrated the superior performance of proposed algorithm.

## Limitations and Future Work

Since our algorithm is proposed for integrating sequence data with microarray measurement of miRNA transfection, target prediction can be carried out only for the miRNAs, for which both types of data are available. Since microarray measurements of genome-wide miRNA transfection are not yet available, it is still infeasible to conduct genome-wide prediction using this algorithm. However, as miRNA transfection becomes increasingly popular and indispensible for miRNA target identification, the need for integrating the two data types is highly desirable. In an effort to provide prediction results, we retrieved around 20 miRNA over-express microarray data From GEO database. The prediction result can be found in http://expmicro.cbi.utsa.edu.

The subsequence work of this paper will focus in two aspects, which are, firstly, continue the predictions for more miRNAs once the two types of data are accessible and secondly improve the mathematical model to further increase the performance.

## Competing interests

The authors declare that they have no competing interests.

## Authors contributions

HL, SJG, and YH conceived the idea. HL, YC, YH worked out the detailed derivations. HL, DY, and LZ, implemented the algorithm and performed the prediction. HL, DY, YH wrote the paper. brodersen2009revisiting
